# 3D-Printed Gastroretentive Tablets Loaded with Niclosamide Nanocrystals by the Melting Solidification Printing Process (MESO-PP)

**DOI:** 10.3390/pharmaceutics15051387

**Published:** 2023-04-30

**Authors:** Juan Pablo Real, Daniel Andrés Real, Lucía Lopez-Vidal, Bruno Andrés Barrientos, Karen Bolaños, Mariano Guillermo Tinti, Nicolás Javier Litterio, Marcelo Javier Kogan, Santiago Daniel Palma

**Affiliations:** 1 Unidad de Investigación y Desarrollo en Tecnología Farmacéutica (UNITEFA), CONICET, Haya de la Torre y Medina Allemde, Córdoba X5000HUA, Argentina; juan.real@unc.edu.ar (J.P.R.);; 2 Departamento de Ciencias Farmacéuticas, Facultad de Ciencias Químicas, Universidad Nacional de Córdoba, Haya de la Torre y Medina Allende, Córdoba X5000XHUA, Argentina; 3 Departamento de Química Farmacológica y Toxicológica, Universidad de Chile, Santos Dumont 964, Santiago 8380494, Chile; daniel.real@ciq.uchile.cl (D.A.R.);; 4 Advanced Center of Chronic Diseases (ACCDiS), Universidad de Chile, Santos Dumont 964, Santiago 8380494, Chile; 5 Center for Studies on Exercise, Metabolism and Cancer (CEMC), Laboratory of Cellular Communication, Program of Cell and Molecular Biology, Faculty of Medicine, Institute of Biomedical Sciences (ICBM), Universidad de Chile, Santiago 8380453, Chile; 6 Facultad de Ciencias Agropecuarias, IRNASUS CONICET, Universidad Católica de Córdoba, Córdoba X5016DHK, Argentina

**Keywords:** MESO-PP 3D printing, nanocrystals, printlets, niclosamide, gastroretentive

## Abstract

Niclosamide (NICLO) is a recognized antiparasitic drug being repositioned for *Helicobacter pylori*. The present work aimed to formulate NICLO nanocrystals (NICLO-NCRs) to produce a higher dissolution rate of the active ingredient and to incorporate these nanosystems into a floating solid dosage form to release them into the stomach slowly. For this purpose, NICLO-NCRs were produced by wet-milling and included in a floating Gelucire l3D printed tablet by semi-solid extrusion, applying the Melting solidification printing process (MESO-PP) methodology. The results obtained in TGA, DSC, XRD and FT-IR analysis showed no physicochemical interactions or modifications in the crystallinity of NICLO-NCR after inclusion in Gelucire 50/13 ink. This method allowed the incorporation of NICLO-NCRs in a concentration of up to 25% *w*/*w*. It achieved a controlled release of NCRs in a simulated gastric medium. Moreover, the presence of NICLO-NCRs after redispersion of the printlets was observed by STEM. Additionally, no effects on the cell viability of the NCRs were demonstrated in the GES-1 cell line. Finally, gastroretention was demonstrated for 180 min in dogs. These findings show the potential of the MESO-PP technique in obtaining slow-release gastro-retentive oral solid dosage forms loaded with nanocrystals of a poorly soluble drug, an ideal system for treating gastric pathologies such as *H. pylori.*

## 1. Introduction

Drug repositioning, also known as drug repurposing reprofiling or re-tasking, is a strategy in which drugs that have already been approved and studied are clinically tested for therapeutic purposes other than the initial ones [1]. Given the emergence of bacterial resistance and the need for new compounds useful against infectious diseases, drug repurposing is an excellent alternative since the risks and costs of development to reach the market are reduced due to the availability of preclinical data [2]. 

*Helicobacter pylori* is a gram-negative helicoidal bacterium capable of growing in the mucous layer lining the interior of the human stomach, causing unfavorable physiological and morphological changes. This microorganism, present in the guts of more than half of the world’s population [3], causes chronic active gastritis that, in a significant minority, triggers gastroduodenal diseases, peptic ulcer being the most recognized. Additionally, this infection is also considered a type I carcinogen. Epidemiological studies have shown that individuals infected with *H. pylori* have a higher risk of gastric adenocarcinomas [4], with non-cardia gastric cancer and gastric mucosa-associated lymphoid tissue lymphoma (MALT) [5] being the most prevalent. Its high prevalence, its carcinogenic potential [6], and the appearance of multidrug-resistant (MDR) strains have led the World Health Organization (WHO) to include this bacterium in a list of twelve priority pathogens, on which it is necessary to work in search of new treatments to eradicate them [7]. 

Niclosamide is an anthelmintic, included in the list of essential drugs [8], which has been safely used since 1962 in millions of human and animal patients for the oral treatment of intestinal parasites [9]. While this drug was believed to act by inhibiting oxidative phosphorylation, in recent years, evidence has accumulated on its ability to inhibit and regulate multiple biological pathways and processes. These characteristics have made it a promising candidate for repositioning in the treatment of other conditions, such as cancer [10], viral [11,12] and bacterial infections [13], including *Helicobacter Pylori*. In a previous work performed by Tharmalingam et al. [14], it was verified that Niclosamide was stable at acid pH, being effective, in vitro and in vivo, against antimicrobial forms of *H. pylori* with a Minimum Inhibitory Concentration (MIC) and a Minimum Bactericidal Concentration (MBC) of 0.25 µg/mL and 0.5 µg/mL, respectively. Despite the accumulated evidence that positions Niclosamide for use in the treatment of *H. pylori*, this drug belongs to the class II of the biopharmaceutical classification system (BCS), being very low in solubility and dissolution in aqueous media, especially at acidic pH [15]. To obtain a successful antimicrobial treatment for *H. pylori* infection, drugs must be stable and active at acidic pH and have the capacity to penetrate the gastric mucosa to reach and maintain the minimum concentrations (MIC and MBC) in this region. In other words, to make more effective use of Niclosamide, it is necessary to carry out additional work to improve their dissolution profiles in acid pH, to increase their concentration, and then load them in pharmaceutical forms capable of prolonging their residence time in the stomach [16]. 

Multiple strategies have been used to improve drug dissolution and solubility profile, such as inclusion complexes with cyclodextrins [17,18], solid dispersions using hydrophilic polymers [19], use of cosolvents [20], liposomes [21], self-emulsifying drug delivery systems [22], among others. Especially nanotechnology has been widely studied to improve the properties of niclosamide with the aim of repositioning it, especially for the treatment of cancer [10]. The formulation of nanocrystals, polymeric nanoparticles, lipid nanoparticles, nanofibers, micelles, and carbon nanoparticles has been explored. The reduction of particle size to the nanometer scale increases the surface area giving the system differential properties, such as improved apparent saturation solubility (as described in the Ostwald–Freundlich equation), increased dissolution rates (Noyes–Whitney equation), and especially in the case of systems, such as nanocrystals, increased bioadhesive to surface membranes (due to the increase in the number of contact points) [17,23,24,25,26]. In particular, nanocrystals, defined as submicron particles composed of 100% stabilized drugs in a crystalline state, emerge as one of the most promising approaches for improving the in vivo performance of orally administered class II drugs [27]. Among their advantages over other nanoparticulate systems, such as lipid or polymeric systems, are their high loading capacity (mainly justified by the absence of carrier materials), long stability, and solvent-free and easily scalable production methods [28]. 

However, the main disadvantage of nanocrystals (NCRs) is their difficulty in being formulated into solid dosage forms. NCRs have unfavorable flow properties for processing. In turn, the application of external forces (as in granulation and compaction) can lead to irreversible aggregation of the particles when the proportion of NCRs in the powder mixture is high. Additionally, the low density of NCRs also limits their formulation in hard gelatin capsules. In other words, the formulation of NCRs in solid dosage forms is a challenge, especially at high concentrations [29,30,31]. 

To overcome these challenges, in previous works, our group has incorporated albendazole NCRs in solid pharmaceutical forms loaded up to 50% [32], using the 3D printing method, patented by the University of Cordoba, MESO-PP (an acronym for melting solidification printing process) [33]. This method, based on the extrusion of semi-solids by syringe of materials that melt in the range of 40 to 60 °C, allows not only to load of high percentages of NC without using extreme forces but also has advantages inherent to 3D printing, such as (a) creating geometries that are not achievable by traditional methods, such as hollow or porous structures that allow solids to float [34,35], and (b) modulating release rates using different carrier materials, such as Gelucire (fatty polyethylene glycol esters) [36]. 

The 3D printing technology has already been used to manufacture 3D printed niclosamide-loaded tablets using Affinisol™ 15 LV, PEG 6000 and hydroxypropyl-β-cyclodextrin (HP-β-CD) as polymeric supports. To achieve this, a direct powder printing technique (DPE) was used that avoids obtaining the typical filaments used for FDM (fused deposition modeling) printing [37]. An important difference between this technique and MESO-PP is that it uses high temperatures (180 °C) to achieve the prints. To our knowledge, the use of 3D printing to obtain solid dosage forms loaded with niclosamide nanosystems has not been documented. Nor have any papers been found documenting the production of gastroretentive niclosamide-modulated release systems.

**Figure 1:**
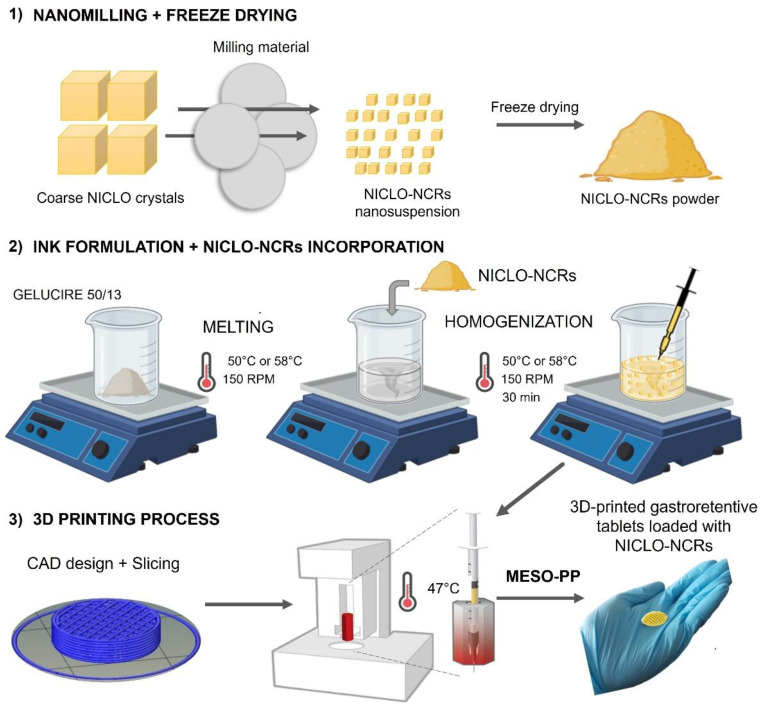
Workflow for Niclosamide nanocrystals formulation and loading into floating 3D printed tablets by the Melting Solidification Printing Process (MESO-PP).

The objective of the present work was to develop NICLO-NCRs with the ability to enhance dissolution profiles and then use the MESO-PP 3D printing technique to incorporate these NCRs into floating 3D printed tablets (Figure 1) with the ability to release them slowly into the stomach.

## 2. Materials and Methods

### 2.1. Materials

Niclosamide (NICLO), poloxamer 188 (P188) and Gelucire 50/13 were obtained from Sigma-Aldrich (St. Louis, MO, USA). The milling process used the yttria-stabilized zirconia beads (Zirmil^®^ Y- Saint-Gobain, Herzogenrath, Germany) of 0.1 mm. In the printing process, 1 mL syringes (Bremen, Germany) and 21 gauge cut metal needles (Lhaura, Bogotá, Colombia) were used. Ultrapure Milli-Q water (18.2 MΩ at 25 °C) was filtered with a pore size of 0.22 µm. Cell culture-treated plates and flasks were purchased from Corning Costar (Glendale, AZ, USA). Penicillin/streptomycin (Thermo Fisher Scientific, Waltham, MA, USA), FBS (Thermo Fisher Scientific). (3-(4,5-dimethylthriazol- 2-yl)-5-(3-carboxymethoxyphenyl)-2-(4-sulfophenyl)-2H-tetrazolium inner salt) (MTS) and phenazine methosulfate (PMS) were obtained from Promega (Madison, WI, USA). Other reagents (analytical grade if not specified otherwise) were purchased from Thermo Scientific. 

### 2.2. Niclosamide Nanocrystals Manufacturing

#### 2.2.1. Nanomilling

The NanoDisp^®^ mill (Córdoba, Argentina), already described in previous works [20,21,22], was used to carry out this process. The NICLO milling process was carried out at 15 °C, for 6 h, with a speed of 1600 rpm, using 0.1 mm zirconium microspheres, P188 and NICLO in a 1:1 ratio (2 g and 2 g) and Milli-Q water up to a final volume of 120 mL. The hydrodynamic particle diameter (size) and polydispersity index (PDI) of the obtained nanosuspensions were evaluated every 2 h.

#### 2.2.2. Freeze Drying and Moisture Content 

The obtained nanosuspension was frozen at −20 °C (overnight), and the samples were freeze-dried for 72 h using a Labconco Freezone 2.5-L benchtop freeze dryer (Kansas City, MI, USA). The amount of moisture in the powder was measured using an OHAUS M45 moisture analyzer (D.F.; Mexico) that uses halogen heating. The analysis of the moisture content of the samples was carried out right after the freeze-drying process. 

### 2.3. Particle Size and Polydispersity Index 

In order to be characterized in terms of size and PDI, 10 mg of the obtained niclosamide nanocrystals (NICLO-NCR) were suspended in 2.5 mL of water and shaken by hand for 1 min. The diluted samples (1:100) in Milli-Q water were measured in triplicate using a Zetasizer^®^ Nano ZS 90 (Malvern Instruments, Malvern, UK).

### 2.4. Nanoparticles Tracking Analysis (NTA)

The concentration of nanocrystals of different sizes after redispersion was determined by nanoparticle tracking analysis (NTA) using a NanoSight NS300 instrument (NanoSight, Amesbury, UK). For this purpose, a procedure similar to that used for the DLS measurement was used, using 10 mg of powders added in 2.5 mL of water, which was then diluted with MilliQ water at a ratio of 1:20 prior to the measurements

### 2.5. Scanning Electron Microscopy (SEM) and Scanning Transmission Electron Microscopy (STEM)

Scanning electron microscopy (FEI Inspect 50-USA with an accelerating voltage of 3–20 kV) at magnifications between 500× and 14,000× was used to analyze the surface morphology and the internal structure of the printlets was sputtered with Au prior to examination. For STEM imaging, the following procedure was followed: 10 μL of nanosuspension (fresh or lyophilized and reconstituted) was pasted onto a copper grid (200 mesh, coated with Formvar). After 2 min, the suspension was removed using filter paper. The grid was washed twice with a single drop of Mili-Q water for 1 min. Each drop was removed with filter paper. Next, a 1% phosphotungstic acid drop was added to the grid to be removed with filter paper at 1 min. Finally, the grid was allowed to dry for 1 h before analysis.

### 2.6. Cell Culture

MTS cell viability assays were performed on two cell lines: (1) AGS (Gastric adenocarcinoma) cell line (ATCC^®^ CRL-1793™) cultured in complete RPMI culture medium, containing 1% penicillin/streptomycin and 10% fetal bovine serum. (2) GES-1 (Immortalized human gastric epithelium) cell line (kindly provided by Dr. Andrew Quest, Universidad de Chile, Chile) cultured in complete DMEM culture medium, containing 1% penicillin/streptomycin and 10% fetal bovine serum. The culture was maintained at 37 °C and 5% CO_2_.

#### Cell Viability MTS Assays 

GES-1 cells or AGS cells (10,000 cells/well) were seeded for 24 h at 37 °C and 5% CO_2_ in a pretreated 96-plate. Then, after removing the medium, the cells were incubated for 24 h in a fresh medium containing the treatments. Finally, following the manufacturer’s recommendations, this medium was replaced by a medium containing MTS/PMS reagent without phenol red. The absorbance of the culture medium at 490 nm was recorded using a microplate reader after 3 h incubation. The cell viability was calculated compared to a non-treated control as live control. 

The treatments were prepared from a standard 10 mM solution in PBS buffer + 0.5% DMSO and diluted in PBS for cell viability. A vehicle control treatment was performed, including 0.5% DMSO in PBS.

Data are represented as mean ± SEM showing four independent experiments with each point performed in quadruplicate. A one-way parametric ANOVA with Dunnet’s posttest was used as statistical analysis to make comparisons of all conditions with the mean control. Statistical analysis was performed using GraphPad Prism V9.3.1.

### 2.7. Ink Formulation

The formulation was carried out similarly to that described in previous works using the following proportions: 75% Gelucire 50/13 and 25% NICLO-NCRs (composed of P188 and Niclosamide in a 1:1 ratio) [32]. First, the desired amounts of Gelucire were added to a beaker. Using a water bath, the powders were melted at 50 °C or 58 °C with continuous stirring at 150 ± 10 RPM. Once melted, the NICLO-NCRs were added. The molten ink, with a final concentration of 12.5% Niclosamide, was kept in agitation for 30 min to be loaded into syringes functioning as printing cartridges finally. The loaded syringes were left to stand for at least 24 h.

### 2.8. Infrared Spectroscopy 

A Fourier Transform Infrared Spectrophotometer (FTIR; Agilent Technologies Cary 630) was used to characterize Gelucire 50/13, Niclosamide, and P188 samples and the different formulated inks. Before analysis, each powdered sample (2 mg) was mixed homogeneously into a fine powder of potassium bromide (KBr). Samples were scanned from 4000 to 400 cm^−1^, and the resolution was 1 cm^−1^. Data analysis was processed using OMNIC^®^ 9.2 software (Thermo Fisher Scientific, Waltham, MA, USA).

### 2.9. X-ray Diffraction

A PANalytical X’Pert ProVR powder X-ray diffractometer (PANalytical BV, Almelo, The Netherlands) was used to obtain X-ray diffraction (XRD) patterns of Gelucire 50/13, Niclosamide, P188 and the different inks loaded with NCR or with physical mixes (PM) of NICLO + P188. The equipment was operated at a 2°/min scan rate in continuous scan mode. The 5°–60° range was scanned at an angle of 2θ at a speed of 0.04° 2θ/s. Data analysis was performed using OMNIC^®^ 9.2 software (Thermo Fisher Scientific, Waltham, MA, USA).

### 2.10. Thermogravimetric Analyses and Differential Scanning Calorimetry 

A Discovery HP TGA (TA Instruments, New Castle, DE, USA) was used for the thermogravimetric analysis. Thermogravimetric analysis (TGA) curves were obtained at 25–350 °C under a dynamic N_2_ atmosphere (50 mL/min) at a heating rate of 5 °C/min. Samples were weighed (~5 mg) and sealed in pierced aluminum pans. Differential scanning calorimetry (DSC) was performed to evaluate the thermal properties of the samples (~5 mg): raw materials, NICLO-NCRs, and loaded and unloaded inks with NCRs or physical mixtures (PM) of NICLO + P188. Samples were weighed and sealed in non-airtight aluminum pans. The equipment used was a Discovery DSC 25P (TA Instruments, New Castle, DE, USA). DSC measurements were performed under a dynamic N2 atmosphere (50 mL/min) at a heating rate of 5 °C/min. The temperature range used was 0 °C to 250 °C. Indium standard (MP = 156.6 °C; Delta H fus = 28.54 J/g) was used for calibration. Data analysis was performed using the TRIOS 5.4 software (TA Instruments, New Castle, DE, USA).

### 2.11. Hot-Stage Microscopy

To observe the behavior of NICLO, Gelucire 50/13, NICLO-NCRs, and loaded and unloaded inks at the formulation and printing temperatures, hot-stage microscopy (OLYMPUS Model BX-51, Shinjuku, Tokyo, Japan) was used. The samples loaded on slides were fixed to a polarized light microscope coupled to the stage, which was heated at a rate of 10 °C/min. The images taken within the temperature range of 30 to 70 °C were analyzed using the INFINITY 6.5.6 software (Ottawa, Canada).

### 2.12. Rheological Analysis

Amplitude sweeps, frequency sweeps, strain recovery tests (Step-Test) and Melting-Solidification simulation of flat-faced cylinders of the samples (diameter of 8 mm and thickness of 2 mm) were evaluated using an oscillatory rheometer (Anton Paar Physica MCR 301, Graz, Austria) with a plate-plate.

### 2.13. Printlet Set Up 

Using a computer-aided design platform (Fusion 360), flat-faced cylinders with a radius of 5.5 and a height of 6 mm were designed. Subsequently, this 3D geometric design file (in STL format) was processed using the Repetier Host (Repetier-Host V 2.1.3, Willich, Germany) in order to create the file (g-code) that is used by the printer to obtain the printlets. A 40% rectilinear infill pattern was used to create this file with filaments arranged at 45° in irregular layers. Prints are completed with a single solid top and bottom layer and a 500 μm solid shell.

### 2.14. Printing Process

The 3D-printed tablets loaded with NCRs (INK + 25% NCRs) or the physical mixture of NICLO + P188 (INK + PM) (not nano) were obtained using the MESO-PP technique [19]. MESO-PP is an extrusion method based on heated syringes. The syringe is placed inside an electrically heated alloy tube to allow the mixture to stabilize at optimal printing temperature (47 °C). Following the instructions housed in a g-code file, the vertical tower of the printer (3-Donor^®^ developed by Life SI, Córdoba, Argentina) moves from right to left at a speed of 4.5 mm/s while the piston presses the plunger. From the syringe (extrusion rate: 0.001 cm^3^/s; printing pressure: 0.02 bar), the mixture gradually settles in a semi-solid state on the print bed (temperature 25 °C) to finally solidify at room temperature. The process is repeated layer by layer until the previously designed three-dimensional image is completed. A short 21-gauge metal needle with an internal gauge of 0.514 mm was used for printing.

### 2.15. Weight/Volume Ratio and Weight Variation 

To evaluate the feasibility of dose adjustment by changing the printlet size, the weight/volume ratio was calculated using samples of 100% to 60% of the original size. Additionally, the weight and dimensions of the prints obtained were used to assess the precision and variability of the printing process. Following the guidelines indicated in USP (USP, 2007), the obtained forms’ weights’ uniformity was evaluated. For this, it was necessary to weigh ten printlets of equal size, calculate the average mass and verify that no more than two individual masses deviate by more than 5% from the average mass. A Vernier caliper (BTA tool) was used to measure the dimensions of the prints. 

### 2.16. Buoyancy and In Vitro Drug Release Evaluation 

In vitro release studies of different printlets and hard gelatin capsules containing niclosamide and NCR were carried out in 900 mL of 0.1 N HCl (pH 1.2 ± 0.5) with and without 2% Tween 80. The hard gelatin capsules were loaded with the calculated equivalent amount of Niclosamide loaded in the printlets. The procedure followed USP guidelines, with the USP II Dissolution Apparatus (AT7 Smart Dissolution Tester, Sotax Corporation, Boston, USA) at 37 ± 0.5 °C and paddle configuration at 50 rpm stirring. Samples of 3 mL were taken at predetermined intervals (10, 20, 30, 40, 40, 50, 60, 60, 90, 120, 180, 180, 240, 300, 360, 360, 540, 720 and 1440 min) and, after filtration, dissolved Niclosamide was quantified using a spectrophotometer at 330 nm. The visual evaluation determined the total floating time (time during the printlets remain floating) during the dissolution test.

### 2.17. In Vivo Gastroretention

To evaluate the gastroretention of the printlets into the stomach, printlets were formulated, including barium sulfate, for contrast enhancement. Then, they were administered orally to a dog, and radiographs were taken at 30 and 180 min. Before sample administration, the animal fasted for 8 h, and water was administered ad libitum. This trial was performed according to the bioethical protocols of the Universidad Católica de Cordoba (Argentina). 

### 2.18. Statistical Analysis

Each result was expressed with its mean value ± standard error (SD). GraphPad Prism 7.0 software (GraphPad Software, CA, USA) was used for statistical analysis. An analysis of variance test was used for statistical comparison/analysis. A *p*-value of 0.05 or less was considered statistically significant. 

## 3. Result and Discussion

### 3.1. Formulation and Characterization of Niclosamide Nanocrystals 

Based on previous results obtained with other drugs [32,38], P188 was selected as the stabilizing agent for the 0.1 mm zirconium sphere-assisted milling performed with the NanoDisp^®^ laboratory-scale mill (Córdoba, Argentina). During the milling time of 6 h, it was observed that throughout the process, the particle size and PDI of the formulation followed a decreasing trend (Table 1), finally obtaining suspended submicron particles of the drug with a particle size of 188.5 nm with a PDI of 0.186, confirming the obtaining of a monodisperse nanosuspension (NS). 

Although the NS was designed to be converted into dispersible solids that would then be incorporated into inks for printing, we studied for 35 days whether, prior to drying, the particle size and PDI remained constant. This analysis was carried out on NS stored in a refrigerator at 4 °C and on NS exposed to room temperature (25 °C). As detailed in Table 2, throughout this monitoring, it could be observed that although in both conditions there is a slight increase in particle size, more marked at room temperature, the NS remains with submicron and monodisperse particle sizes after one month of storage.

After milling, the NS was subjected to freeze-drying to eliminate the water content and obtain the nanocrystals. The obtained powder was measured for size and PDI to evaluate the redispersion capacity of the NCRs, obtaining particles with an average Z-average of 357.0 nm and a PDI of 0.304, which remained without significant changes during the 21 days (Table 3) that it was monitored by dynamic light scattering (DLS). The moisture content of the NCR obtained in the drying process was also determined. The values found ranged from 1.9% to 2.3%.

**Figure 2:**
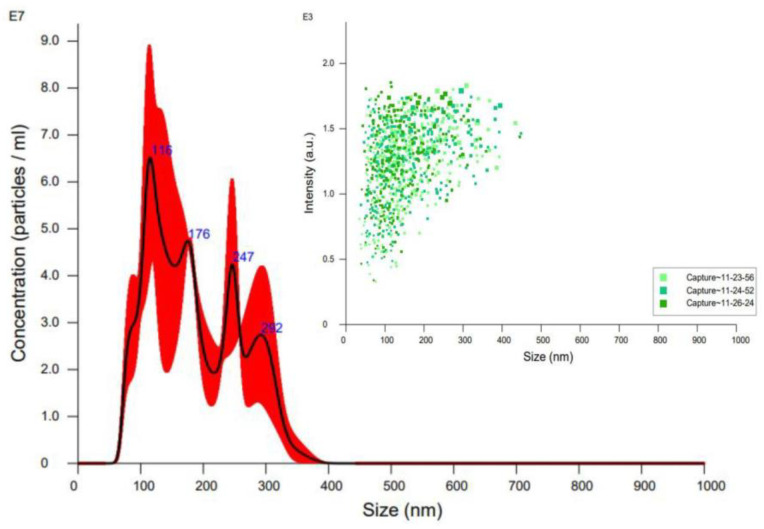
Nanoparticles Tracking Analysis of Redispersed NICLO-NCRs.

Although DLS is the most widely used technique for particle size measurement due to its simplicity, accessibility and non-destructive nature, it has inherent limitations of intensity-biased detection. It is important to note that the z-average from DLS is a hydrodynamic parameter sensitive to small changes in the sample, such as aggregates or dust particles. To address this problem, we decided to use other techniques to verify the size determined by DLS. First, we use nanoparticle tracking analysis (NTA) which, like DLS, uses the diffusion coefficient to measure particle size with a different principle. While in DLS, the diffusion coefficient of the particles is calculated from the change in light intensity, NTA uses the motion of individual particles captured in successive optical video images to calculate the diffusion coefficient. The change in the detection principle leads to differences in how particle sizes are reported, especially in measured particle quantities. As shown in Figure 2, most redispersed NCRs retain a particle size below 200 nm, and no crystals above 400 nm are observed.

Moreover, scanning electron microscopy (SEM) and scanning transmission electron microscopy (STEM) can image particles and even measure the width of individual particles in solid and re-dispersed states, respectively. This is important because the hydrodynamic size measured by DLS considers the scattering size of a hypothetical rigid sphere. However, macromolecules may not be spherical; they are dynamic and can aggregate and solvate.

**Figure 3:**
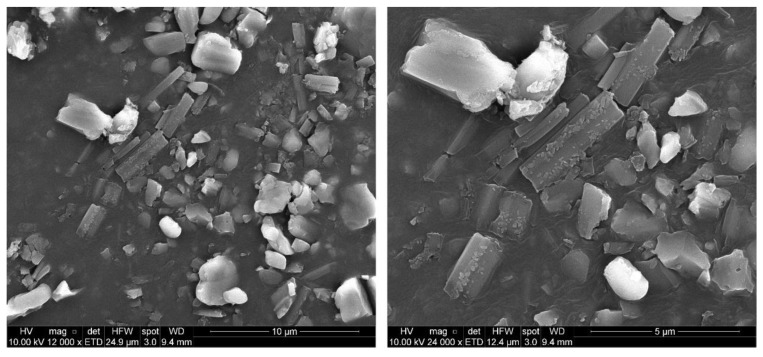
Scanning Electron Microscopy of raw Niclosamide powder.

**Figure 4:**
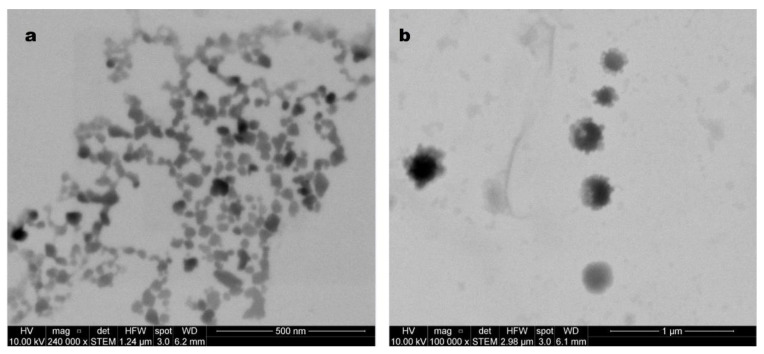
STEM analysis of redispersed NICLO-NCRs freeze-dried powder. (**a**) Size distribution of NCRs. (**b**) Detail of the presence of poloxamer on the surface of the NCRs.

As seen in Figure 3, SEM images of raw NICLO reveal a crystalline solid irregular in size and geometry. However, after the milling and drying process, the STEM micrographs (Figure 4) of the redispersed nanocrystals in water show a population of particles with different geometries, typical of a milling process, with a relatively homogeneous size distribution of less than 250 nm in almost all cases. Moreover, in Figure 4b, it is possible to appreciate some particles that partially or entirely coat the NCRs, probably corresponding to P188, a factor that favors the stability of the crystals, avoiding clustering after their redispersion.

In order to study the gastrointestinal toxicity of NICLO exposure, we studied the viabilities of GES-1 cells after exposure to NICLO and NICLO-NCRs. The variability was quantitatively measured using the MTS assay, which reflects the mitochondrial activity of the cells. Generally, the viability loss depends on the dose and exposure period. In this case, the cells were treated in both cases for 24 h in an increasing dose range from 1 to 25 µM Niclosamide. 

**Figure 5:**
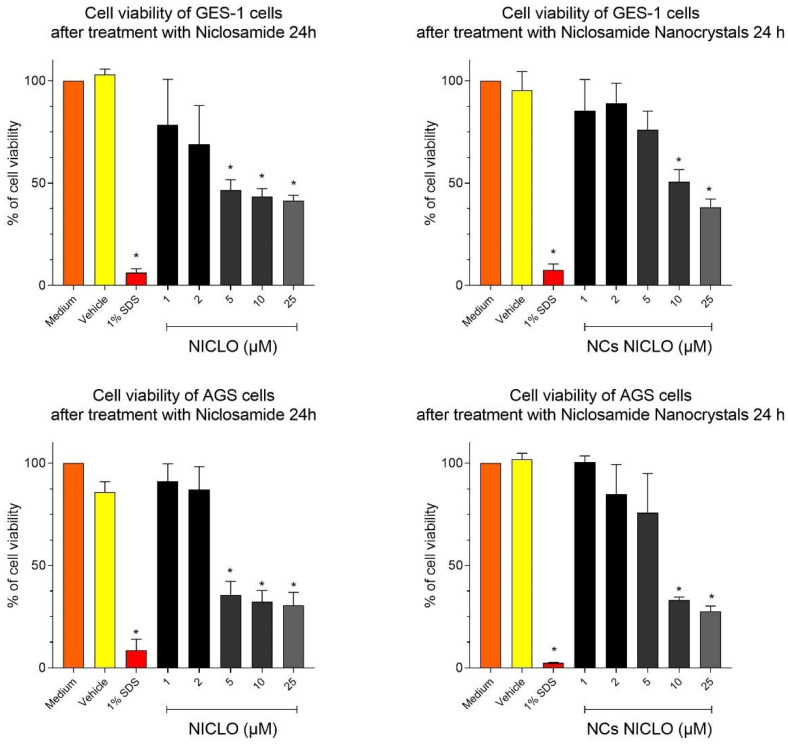
Cell viability assays in the GES-1 cell line (top) and AGS cell line (down). Data are represented as mean ± SEM showing the results of four independent experiments with each point performed in quadruplicate. Statistical analysis was performed using a parametric one-way ANOVA with Dunnet’s post-test comparing all conditions with the medium control. Statistical significance is indicated with * denoting *p* < 0.001 or *p* < 0.0001, respectively.

The results (Figure 5 top) show that after a 24 h treatment, the cell viability of GES-1 decreases with increasing concentrations of untreated Niclosamide, which is significant from 5 µM, equivalent to 1.6 µg mL^−1^. When the culture was performed under exposure to NICLO-NCRs, the loss of viability of GES-1 cells was not significant up to concentrations of 10 µM (3.2 µg mL^−1^), which would indicate that the reduction of particle size not only does not increase toxicity but in some way favors viability. This data is relevant since the nanocrystal formulation aims to increase the concentration levels reached when Niclosamide is administered orally.

The AGS cell viability assay was performed with the intention of observing whether, in gastric cancer cell lines, a higher uptake and, therefore, toxicity was observed with respect to cell lines of non-cancerous origin. As can be seen in Figure 5, AGS cell viability showed very similar behavior to that observed in GES-1 cells upon exposure to untreated Niclosamide and NICLO-NCRs. NCRs again generated a protective effect up to certain concentrations of niclosamide, which we believe may be due to the presence of P188. The ability of P188 to form aggregates in which free niclosamide can be trapped is documented [39]. This phenomenon would decrease the amount of free niclosamide available in the medium, thus decreasing toxicity to some extent.

### 3.2. Ink Formulation and Physicochemical Characterization

As already described in Section 2.7 for the formulation of the printing inks, NICLO-NCR was mixed at a ratio of 25% *w*/*w* in solid form with known and melted amounts of Gelucire 50/13. The mixture in a beaker was kept under heat and constant stirring for 30 min. To evaluate the influence of the melting of the P188 contained in the NCRs, the ink was formulated at 50 and 58 °C. Each homogenized molten mixture was loaded into syringes and left to stand for 24 h for subsequent printing and characterization (Figure 1). 

In this work, four inks were formulated and characterized: NICLO-NCRs and Niclosamide mixed with raw P188 (Physical Mixture, PM) formulated at 50 and 58 °C (Table 4). Each of these inks underwent Fourier transform infrared spectroscopy (FTIR), X-ray diffraction (XRD), thermal analysis (TGA and DSC), Hot Stage Microscopy (HSM), and finally, an analysis of their rheological behavior.

#### 3.2.1. Fourier Transform Infrared Spectroscopy (FTIR)

**Figure 6:**
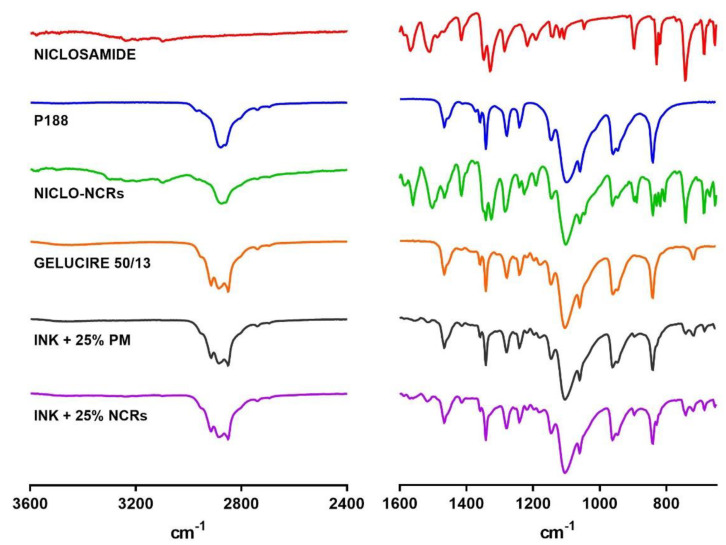
FTIR analysis of NICLO, P188, NICLO−NCRs, GELUCIRE 50/13, and INKs loaded with NCRs or PM (not nano).

FTIR is usually used to evaluate the chemical structure of drugs, excipients, and the resulting formulations to rule out possible chemical interactions. The characteristic infrared bands of halogen-containing organic compounds, such as Niclosamide containing C─Cl bond, are seen between 800 and 400 cm^−1^. As shown in Figure 6, these and other characteristic NICLO peaks were present in both NCRs-NICLOs and the different formulated inks. These findings indicate that no chemical interactions between the drug, stabilizer and Gelucire occurred during the different manufacturing and formulation processes.

**Figure 7:**
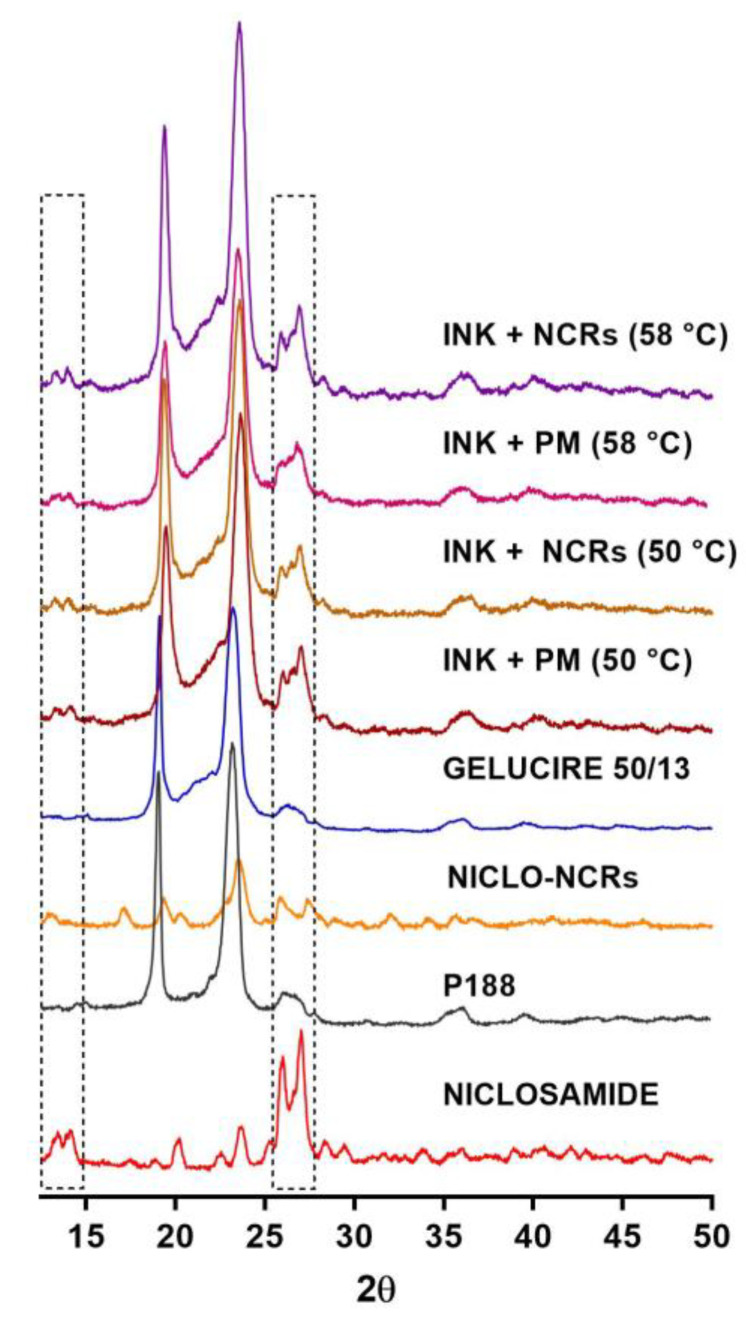
X-ray diffraction spectra of NICLO, P188, NICLO-NCRs, GELUCIRE 50/13, and INKs loaded with NCRs or PM formulated at 50 °C or 58 °C. Characteristic peaks of crystalline NICLO were highlighted using dotted lines.

This observation was confirmed by XRPD (Figure 7). Powder X-ray diffraction of the drug, the untreated excipients, the NCRs-NICLO and the different inks were performed, and the signals assigned to each pure component were observed unchanged. This confirms that the drug does not change its crystallinity when incorporated into the printing inks.

#### 3.2.2. Thermal Analysis: TGA and DSC

**Figure 8:**
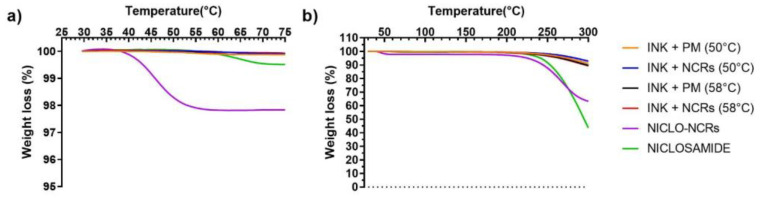
TGA analysis of NICLO, NICLO-NCRs, and INKs loaded with NCRs or PM (not nano) formulated at 50 °C or 58 °C. (**a**) 25 °C to 75 °C temperature range. (**b**) 25 °C to 300 °C temperature range.

**Figure 9:**
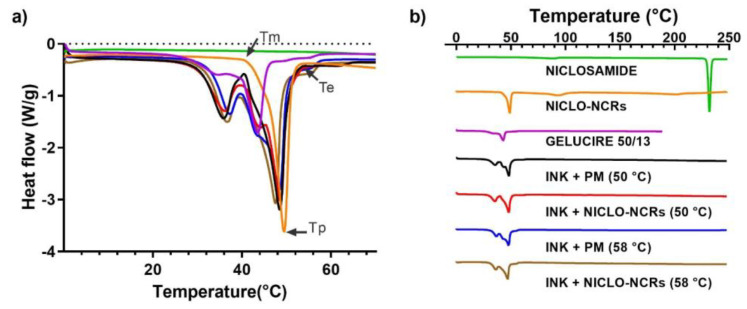
DSC analysis of NICLO, NICLO−NCRs, GELUCIRE 50/13 and INKs loaded with NCRs or PM (not nano) formulated at 50 °C or 58 °C. (**a**) Data between 0 °C and 70 °C. (**b**) Data between 0 °C and 250 °C.

The thermal TGA and DSC analyses performed for the inks and raw materials (NICLO, P188, and Gelucire) are shown in Figure 8 and Figure 9, respectively. The results of the TGA analysis showed that in the formulation range (50 and 58 °C) and MESO-PP printing temperature (47 °C), no significant mass losses were observed, except for NICLO-NCRs, which accumulated a mass loss of 2% at 50 °C. This percentage corresponds to the moisture content of the freeze-dried nanocrystals. It should be noted that this initial mass loss remained constant up to temperatures above 200 °C and that this phenomenon was not observed in the Niclosamide or the formulated inks, which had weight losses of less than 0.5% up to 200 °C. At elevated temperatures above 250 °C, thermal degradation of Niclosamide and the NICLO-NCRs was observed, demonstrating the importance for this drug of using 3D printing methods that do not use extreme temperatures.

DSC curves of multicomponent materials, such as Gelucires and MESO-PP inks, may show broadened enthalpic peaks with an inflection temperature at the beginning of the extrapolation (Tm), a peak temperature (Tp) and a return to baseline temperature (Te), far apart (Figure 9a). DSC curves of raw Niclosamide, NICLO-NCRs, Gelucire 50/13, and the formulated inks can be seen in Figure 9. The curves of Niclosamide exhibited a characteristic melting point at ~230 °C, which could not be observed for NICLO-NCRs due to drug dissolution in P188 before reaching 230 °C. The diffractogram obtained for Gelucire 50/13 was similar to that previously reported, exhibiting a minimal prior transition and a main endothermic event with a peak at 43.5 °C. 

As for the formulated inks, all showed similar behavior, with a slight inflection point at 30 °C, equivalent to that shown by Gelucire, and two pronounced endothermic melting peaks at 37 °C and a second one at 47.5–48.5 °C for those formulated at 58 °C and 50 °C, respectively. The second endothermic peak in the case of the inks formulated at 50 °C is the result of the overlapping of the melting peaks of G 50/13 and P188 present in the nanocrystals, something that can be seen in the shoulder present at 43.5 °C observed in the 50 °C Nano Ink and absent in the 58 °C Nano Ink. In the inks formulated at 58 °C, the mixture of G50/13 with P188 produces a shift in the melting peaks.

This thermal analysis is relevant in printing processes based on hot extrusions, such as MESO-PP. Before the MESO-PP printing process, the material is solid inside the syringes, acquiring a semi-solid state with a suitable viscosity when exposed to the printer’s heating. At the printing temperature, the material must acquire a sufficiently low viscosity to be extruded at low pressure through a needle and high enough to form a filament without collapsing on the printing platform. Based on the DSC curves obtained from the inks, the printing temperature was defined as 47 °C, a temperature slightly lower than the second endothermic peak observed in the inks, which ensures that the material is not entirely melted. 

#### 3.2.3. Hot Stage Microscopy 

The Hot Stage Microscopy (HSM) was used to visually evaluate the above-described thermal behaviors of the different materials and inks. Within the temperature range analyzed (30–70 °C), several vital temperatures, such as the printing temperature of 47 °C and the two formulation temperatures of 50 and 58 °C, were chosen to represent the behavior. 

**Figure 10:**
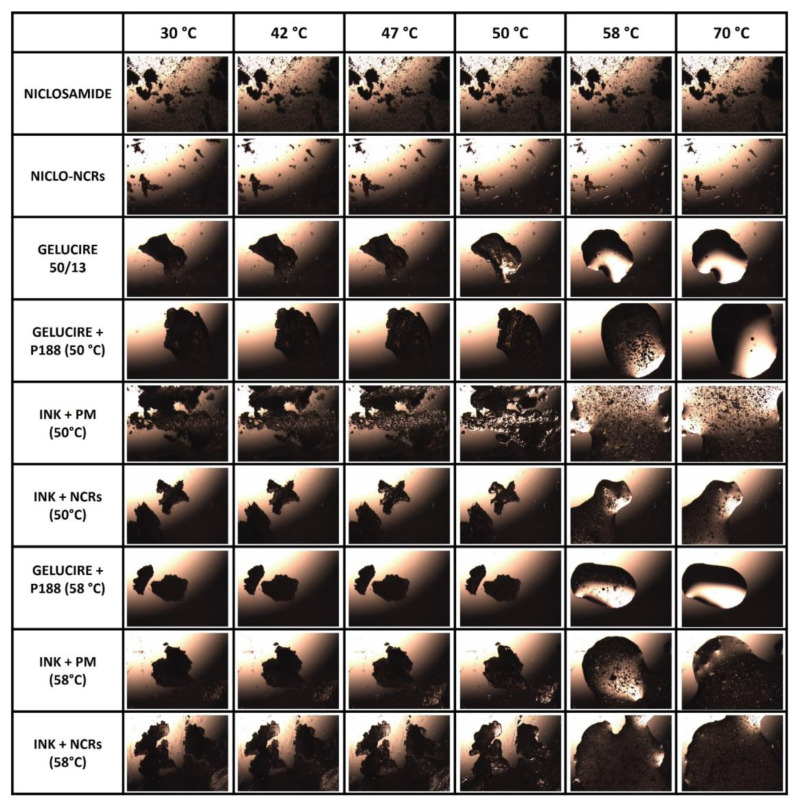
HSM photomicrographs at 30 °C, 42 °C, 47 °C, 50 °C, 58 °C and 75 °C. Heating ramp 10 °C per min (40×).

As shown in the first row of Figure 10, the NICLO structure remains unchanged throughout the observed temperature range. When analyzing the structure of NICLO-NCRs, it could be observed (Row 2) that they remain unchanged up to temperatures above 50 °C, from which the embedded in the molten poloxamer can be observed. Concerning the inks, the HSM technique confirmed the semi-solid state at 47 °C and the influence of nanometrization and formulation temperature on suspended particles’ quantity, size and distribution. The particle size reduction and the number of agglomerates as a consequence of nanometrization are seen in the comparative analysis of the images at different temperatures of the Nano and non-Nano formulations. On the other hand, the influence of the formulation temperature and the mixture of G50/15 and P188, previously discussed, is seen in comparing the image at 58 °C of the white Gelucire and P188 ink formulated at two different temperatures. A large number of scattered particles (dark spots) corresponding to P188 can be observed in ink formulated at 50 °C. In the short exposure time at 58 °C in the HSM test, P188 fails to melt and mix entirely with G50/13. In ink formulated at 58 °C, the number of dark spots decreases significantly, which implies that some of the P188 have been premixed with G50/13. This visually confirms what we had previously analyzed by DSC.

#### 3.2.4. Rheological Behavior 

Finally, we decided to characterize the formulated inks from a rheological point of view. From this perspective, for the ink to be suitable for use with the MESO-PP method, it must exhibit a high viscosity at rest (inside the syringe) and have a pseudoplastic behavior. This implies that the ink must be held in the syringe and not flow until sufficient pressure is applied to the cylinder. When pressed, the material must adopt a shear thinning behavior, i.e., a drop in viscosity sufficient to allow it to extrude through the needle. Finally, when exiting the printing nozzle, the ink must have a rapid recovery of viscosity to avoid further flow once deposited on the printing platform and be able to serve as a solid base for the successive layers, supporting their weight.

The rheological behavior of the Gelucire and the different inks was evaluated employing four consecutive dynamic rheology tests. In these tests, a slight deformation or strain rate is applied in an oscillatory manner on the material to be analyzed, measuring the amplitude of the shear response and the phase angle between the shear stress and the deformation. In viscoelastic materials, whose response is the result of the sum of the response of both components, this type of test allows determining the proportion between the elastic and viscous components of a material, which are represented in the Storage Modulus (G’) and Loss Modulus (G″), respectively. From the value of these moduli and the ratio between them, we can analyze the material’s structure (whether stronger or weaker) and its predominant behavior at different strain levels. When the predominant behavior is viscous (more like a liquid), G″> G’, while when the predominant behavior is elastic (more like a solid), precisely the opposite occurs (G’ > G″).

**Figure 11:**
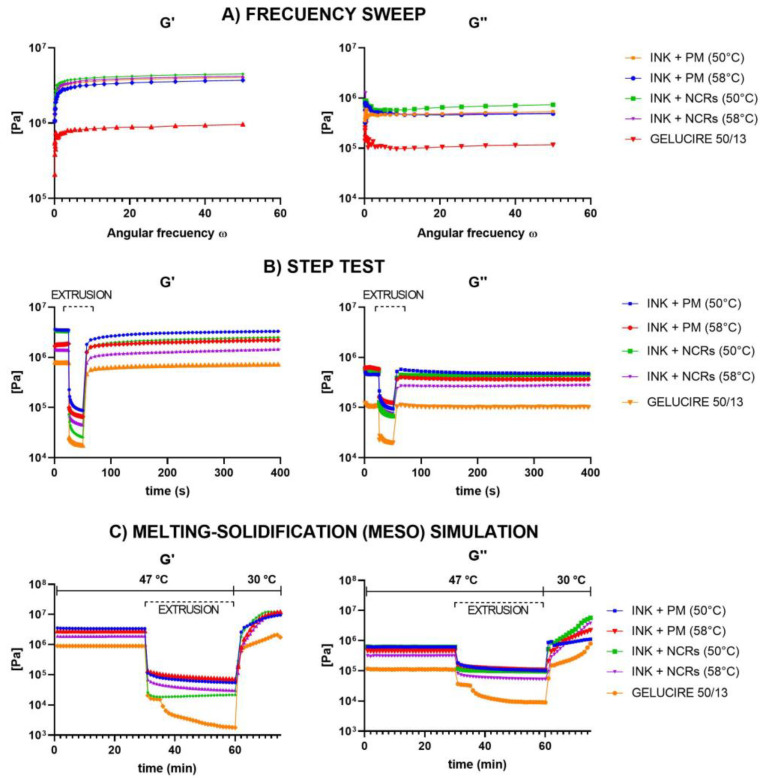
Frequency rheological analysis of GELUCIRE 50/13 and INKs loaded with NCRs or PM formulated at 50 °C or 58 °C. (**A**) Frecuency sweep test. (**B**) Step test. (**C**) Melting-Solidification (MESO) Simulation.

Since the material’s viscosity and behavior depend on temperature, the tests were initially carried out at 47 °C (printing temperature). First, an Amplitude Sweep was carried out to delimit the Linear Viscoelastic Region (LVR) of the inks, i.e., the stress range in which the Complex Modulus (G* = G’ + G’’), which represents the total resistance of a substance to an applied deformation, remains constant and does not depend on other rheological parameters, such as stress or strain (not shown). Then, a Frequency Sweep (Figure 11A) was performed. In this test, the strain amplitude remains constant (within LVR), and the frequency progressively increases. Through this test, we were able to observe, as expected, that the value of G’ was higher than G″ (which means that in a resting state, the material behaves more like a solid) and that, in turn, the values of the moduli of the inks were higher than those observed with the Gelucire, something that structurally agrees with the addition of a dispersed solid. As we have already observed in the HSM analysis, the number of dispersed solids is higher in the inks formulated at 50 °C and with the presence of nanocrystals, this being reflected in the value of both moduli.

After this analysis, a test known as Step Test was performed. Step tests evaluate the breakdown of a sample’s inner structure under high shear and its subsequent recovery. The following intervals were programmed to carry it out: A first stage with very low shear to simulate behavior at rest at low strain. A second one includes high strain, far beyond the LVR, to simulate the structural breakdown that the inks undergo when extruded. Furthermore, the third and final stage uses the same low strain value as the first interval to simulate structural regeneration at rest. The results of this test (Figure 11B) showed that all the inks, as well as the Gelucire, when subjected to significant stress similar to that of the printer, adopt the required shear thinning behavior, with a decrease in the value of the moduli and inversion in the relationship between them (from G’> G’’ to G’’> G’). In other words, the material adopted a viscous behavior that makes extrusion possible. In the third interval, when the deformation ceases, all the inks showed high percentages of recovery on the values of their modulus, being again the elastic behavior (G’ > G’’) the predominant one. In other words, the ink recovered its viscosity after flowing, which is necessary for the printing process. When comparing the different inks formulated, it can be observed that the Nano-50 °C ink exhibits, especially in the storage modulus, the most abrupt thinning and a high percentage of recovery. 

It should be noted that the analyses performed with the Step Test undergo modifications in the fourth test, which more accurately simulates the physical processes occurring in the MESO-PP method. This last test is similar to the Step Test with the difference that in the recovery process, in addition to canceling the applied strain, the temperature of the material is also abruptly decreased (simulating the exit of the ink from the syringe contained in the thermostated jacket). When analyzing these results (Figure 11C), it is observed that the Nano-50 °C ink, in addition to being the one that suffers the greatest thinning, is the one that has the best recovery speed, quickly reaching values in the modules higher than those present in the resting state in the syringe at 47 °C. 

As a result of all these analyses, the ink formulated at 50 °C was selected for the printing processes because it is the ink with nanocrystals that has the best rheological properties for the printing process.

### 3.3. Printing Process and 3D Printed Tablets (Printlets) Characterization

The printing process was initiated by placing the ink-loaded syringes in the electrically heated alloy tube at the optimum printing temperature (47 °C). Then, the vertical tower of the printer, following the instructions housed in a digital file (g.code), moves in the three axes of XYZ space as a piston presses the plunger of the syringe so that the mixture is gradually deposited in a semi-solid state on the printing bed, where it finally solidifies at room temperature. This process is repeated layer by layer until the desired, designed three-dimensional shape is achieved.

**Figure 12:**
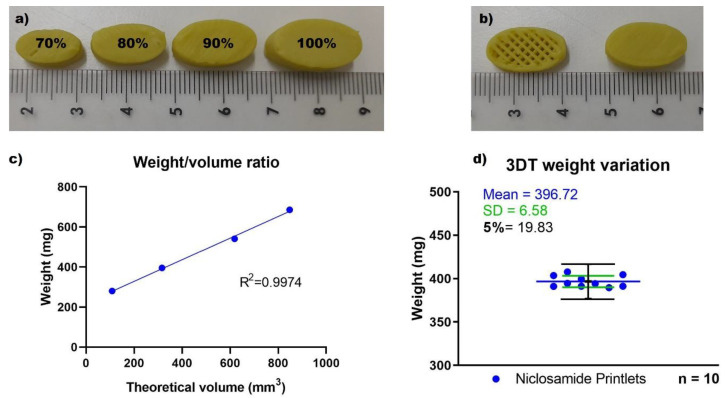
MESO-PP 3D-printed tablets loaded with 25% *w*/*w* of NICLO-NCRs. (**a**) Printlets with different volumes manufactured being 100% an oblong geometry of 6 mm thickness and 10 and 18 mm diameters. (**b**). Selected geometry of 80% volume, printed in full (right) and without the last layer (left) to show the rectilinear internal design (**c**) Weight/volume ratio of printed tablets and (**d**) weight variation of a batch of ten printlets.

Using as a 100% size reference an oblong geometry of 6 mm thickness and 10 and 18 mm of minor and significant diameter, respectively, 3D printed tablets (printlets) of various sizes with an internal filling of 30% were obtained (Figure 12a b). After weighing them and evaluating their buoyancy, It was possible to verify that up to a size of 70% (concerning the design at 100), the printlets maintained their ability to float, with a 99% correlation between the average weight of the tablets and their theoretical volume (Figure 12c). Printlets that were smaller than 70% of their original size lost their ability to float when they had a filling of only 30%. In other work, we have already indicated that this level of filling is crucial for the buoyancy of such systems. These smaller printlets were not included in the framework of this work.

Of the different sizes evaluated, the 80% scaled geometry with an average weight of approximately 400 mg, equivalent to a 50 mg load of Niclosamide, was chosen for the following analysis (Figure 12b). Figure 12d shows the weights obtained from a batch of 10 of these printlets. The average weight of the batch was 396.72 mg, with a standard deviation of 6.58 mg. Outside the range of 5% of the expected weight, no printlets were observed, thus meeting the USP requirement for solid dosage forms of more than 320 mg. The printed tablets also met pharmacopeia specifications for friability and hardness.

**Figure 13:**
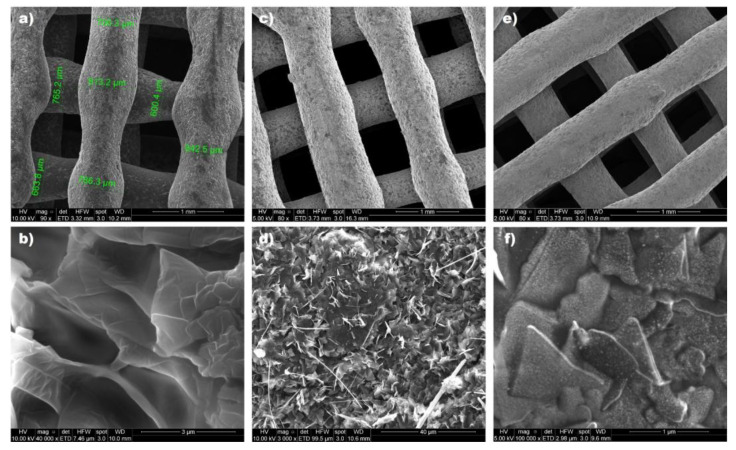
SEM analysis of printlets: Gelucire 50/13 [(**a**,**b**)], INK + PM (50 °C) [(**c**,**d**)], INK + NICLO-NCRs (50 °C) [(**e**,**f**)].

Scanning electron microscopy analysis was performed to examine the structure of the different prints obtained. Figure 13 shows how the individual filaments that make up the internal structure of the printlet are symmetrical and uniformly distributed. This demonstrates the ability of the printer to obtain a network structure, which gives it the ability to float with great precision. Using ImageJ 8.0 software (Maryland, USA), the diameter of the filaments was determined, finding a size varying between ~ 600 and 800 µm, which is larger than the internal diameter of the 21 G needle used (514 µm). This coincides with the predominantly elastic behavior that the materials showed when examined from a rheological point of view. 

When the structure of the printed materials was evaluated in more detail, the Gelucire-containing prints showed flake-like structures (Figure 13b). When the physical mixture was added to the ink, NICLO crystals could be visualized, and acicular shapes could be attributed to the poloxamer (Figure 13d). On the other hand, in the case of the prints with nanocrystals, it was possible to observe the presence of nanocrystals included in the Gelucire 50/13 matrix (Figure 13f). 

**Figure 14:**
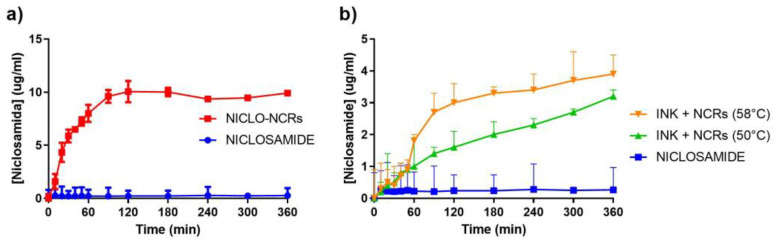
Dissolution profile in HCl 0.1 N (900 mL) of hard gelatin capsules (HGC) including NICLO or NICLO-NCRs (**a**), or printlets loaded with 25% of NCRs formulated at 50 °C and 58 °C (**b**). Apparatus USP-II at 50 rpm and 37 °C.

A dissolution assay in 0.1 N HCl at 37 °C was carried out to evaluate changes in NICLO release profiles in a simulated gastric condition. First, the release of NICLO and NICLO-NCRs included inside hard gelatin capsules (HGC), with an equivalent dose of 50 mg NICLO, was compared. As seen in Figure 14a, the unprocessed drug in the period and conditions evaluated reached a maximum concentration of only 0.36 ± 0.17 µm/mL, a concentration even lower than the MBC that needs to reach the gastric mucosa. The NICLO-NCRs, on the other hand, reached a maximum concentration of 10.01± 0.29 µm/mL, a concentration at least 25 times higher than that reached with the unprocessed drug. Of the maximum concentration achieved with NICLO-NCRs, 80% was reached within the first hour of dissolution. Comparing the dissolution profiles of raw NICLO included inside hard gelatin capsules (HGC) with Printlets formulated at 50 and 58 °C (Figure 14b), it can be observed that the mere presence of these excipients improves the release profile and increases at least 2-fold the maximum concentration achieved. This increase can be attributed to a possible decrease in the interfacial tension of the active pharmaceutical ingredient (API) with the solvent, which is described as to increase in the wettability and solubility of the pharmaceutical ingredients. No differences were observed in the release profiles of the solid dispersions as a function of the temperature at which they were formulated. The time delay in release observed was because the API was included in a lipid excipient (Gelucire) which must be eroded to expose the drug to the solvent. As we have already described, the lower the density of the printlet structure, the higher the release rate. This would explain the improved dissolution profiles observed when NICLO-NCRs are incorporated into the printlets. In this type of system, the process involved in drug release is governed by the combined phenomenon of diffusion or erosion. In the first stage of the process, the sample absorbs the liquid that causes swelling. This phenomenon is favored in Gelucire 50/13, which has a high hydrophilic affinity. The entry of water into the system increases the interstitial space, increasing the mass transfer surface between the particles and the release medium, which is even more favored in the case of printlets loaded with NICLO-NCRs. It should be noted that after 12 dissolution tests under these conditions, all the printlets were floating and retained their geometrical shape.

**Figure 15:**
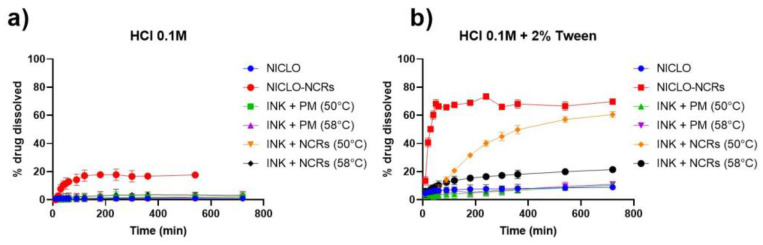
Dissolution profiles of hard gelatin capsules (HGC), including NICLO or NICLO-NCRs, or printlets loaded with 25% of NCRs formulated at 50 °C or 58 °C. Dissolution conditions: 900 mL of HCl 0.1 M without (**a**) or with 2% of Tween 80 (**b**), Apparatus USP-II at 50 rpm and 37 °C.

Taking into account the decisive factor that the surface tension of the dissolution medium has on the rate of dissolution of poorly soluble drugs and considering that the surface tension of human gastric fluid is practically independent of pH and the rate of secretion and that it has a value between 32 and 44 mN/m [40], we decided to repeat the analyses in a 0.1 N HCl medium at 37 °C containing 2% Tween 80 [41,42]. The results obtained (Figure 15b) show an increase in the release profiles of all the systems, being very relevant to what was observed in the printlets loaded with NICLO-NCRs, which managed to increase the NICLO concentration reached levels similar to the NICLO-NCRs loaded in HGC while modulating the release for 12 h. The printlets loaded with NICLO-NCRs were the only ones that achieved total disintegration within the period evaluated under these conditions. In other words, the increase in apparent solubility achieved by NICLO because of its nanometrization and decrease in the medium’s surface tension allowed increasing mass transfer, thus achieving complete erosion and release of the API.

Pistone et al. conducted a study exploring the use of DPE 3D printing to manufacture niclosamide tablets with different polymer-carriers, including Affinisol™ 15 LV, PEG 6000, and hydroxypropyl-β-cyclodextrin (HP-β-CD) [37]. The tablets gradually released niclosamide over 48 h, with those containing HP-β-CD exhibiting a faster release rate. The dissolution profiles of the tablets were examined by subjecting them to simulated gastric and enteric fluids. The study found that the release rates in the gastric medium were similar to those observed in Figure 15b after two hours of dissolution in unprocessed Niclosamide and in printlets containing physical mixtures. In contrast, NCR-loaded printlets exhibited significant differences from unprocessed niclosamide, with the ink formulated at 50 °C displaying the most favorable release profile. As previously discussed in the DSC analysis and the HSM test, the P188 present in the NCR appeared to melt at 58 °C, promoting crystal aggregation and reducing differential properties, such as dissolution rate and apparent solubility. The choice of the ink formulated at 50 °C was reinforced based on these tests. This discovery leads to the conclusion that the nanocrystals cannot be loaded through the method utilized in Pistone et al.’s work. This is due to the fact that the extrusion temperature employed (180 °C) would cause the P188 to melt, resulting in an outcome similar to that observed with ink formulated at 58 °C. Consequently, the use of the MESO-PP technique for producing controlled-release NCR-loaded gastroretentive release systems is further supported.

**Figure 16:**
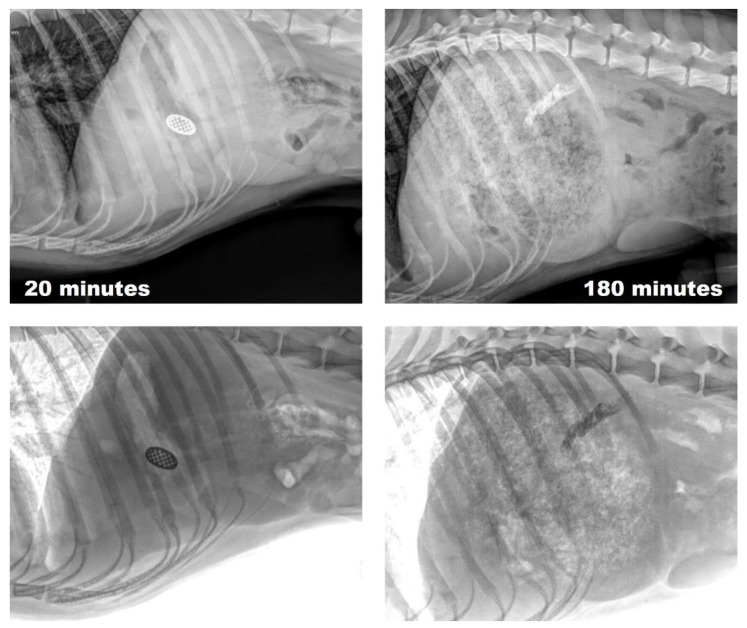
Original (**up**) and negative (**down**) X-ray images of in vivo gastroretention in dogs of floating 3D printed tablets loaded with 25% of Niclosamide nanocrystals. Barium sulfate was used as the contrast agent.

Finally, radiographic studies performed on a single dog showed that the Barium Sulfate-loaded formulation allowed floating for at least 180 min (Figure 16). However, it should be noted that the print lost its shape and changed its position. Within 3 h of ingestion, it is to be expected that the printlet Barium Sulfate-loaded will have eroded and shrunk in size. It should be noted that lipases present in the stomach can increase the speed of this process. On the other hand, the barium sulfate suspension may blur the image, making it difficult to differentiate the exact shape of the printlet. Finally, the printlet may have undergone morphological alterations as a result of gastric motility. Since the erosion and dissolution profile of the G50/13 matrices depends on the API loaded and the medium conditions, further studies are required to evaluate the functionality of the systems in vivo.

## 4. Conclusions

The nanocrystals developed in this work are shown to be an excellent alternative to improve the dissolution profiles of NICLO, a drug that has demonstrated bactericidal activity against *H. pylori* but presents solubility problems in the acidic conditions of the stomach. In this work, NICLO-NCRs have been efficiently incorporated in 3D printed tablets, showing that the MESO-PP technique would be an innovative strategy that would combine the advantages of increased solubility and dissolution produced by the nanometrization of drugs, solve the problems of powder handling presented by nanocrystals, customize the dose, add the functionality of gastroretention and modulate the release of the active ingredient. In conclusion, a solid pharmaceutical form has been obtained with the capacity to produce a local release that a priori could favor the drug’s effectiveness against this bacterium. The in vivo effectiveness of the systems developed based on Gelucire 50/13 requires further research.

## Figures and Tables

**Table 1:** Particle size and PDI were obtained at different milling times.

Time (h)	Z-Average (nm)	PDI
2 h	233	0.226
4 h	198	0.192
6 h	188	0.186

**Table 2:** Particle size and PDI were obtained after different nanosuspension storage times and conditions.

Time (Days)	Refrigerator (4 °C) Z-Average-PDI	Room Temperature (25 °C) Z-Average-PDI
0	188 nm–0.186	188 nm–0.186
7	208 nm–0.186	205 nm–0.170
14	204 nm–0.183	219 nm–0.170
21	224 nm–0.174	215 nm–0.179
28	204 nm–0.164	232 nm–0.147
35	208 nm–0.176	230 nm–0.189

**Table 3:** Particle size and PDI obtained after redispersion of freeze-dried NICLO-NCRs.

Time (Days)	Z-Average	PDI
0	357	0.304
7	356	0.249
14	361	0.289
21	350	0.262

**Table 4:** Formulated inks.

Name	% Matrix Agent (Gelucire 50/13)	% Niclo:P188 (Ratio 1:1)	Niclo:P188 State	Formulation Temperature
INK + PM (50 °C)	75%	25%	Raw powders	50 °C
INK + PM (58 °C)			Raw powders	58 °C
INK + NCR (50 °C)			Nanomilled	50 °C
INK + NCR (58 °C)			Nanomilled	58 °C

## Data Availability

The data presented in this study are available on request from the corresponding author.
